# Molecular Diagnosis and Identification of* Leishmania* Species in Jordan from Saved Dry Samples

**DOI:** 10.1155/2016/6871739

**Published:** 2016-06-15

**Authors:** Nawal Hijjawi, Kalil A. Kanani, Malak Rasheed, Manar Atoum, Mona Abdel-Dayem, Mohammad R. Irhimeh

**Affiliations:** ^1^Department of Medical Laboratory Sciences, Faculty of Allied Health Sciences, Hashemite University, Zarqa 13133, Jordan; ^2^Parasitic and Zoonotic Diseases Department, Communicable Disease Directorate, Ministry of Health, Amman 11855, Jordan; ^3^Princess Iman Center for Research and Laboratory Sciences, King Hussein Medical Center, Jordanian Royal Medical Services, Amman 11121, Jordan; ^4^Faculty of Medicine, Dentistry and Health Sciences, The University of Western Australia, Crawley, Perth, WA 6009, Australia; ^5^Cell & Tissue Therapies Western Australia, Royal Perth Hospital, Perth, WA 6000, Australia

## Abstract

Diagnosis of the endemic cutaneous leishmaniasis (CL) in Jordan relies on patient clinical presentation and microscopic identification. Studies toward improved identification of the causative* Leishmania* species, especially in regions where multiple species exist, and the introduction of these techniques into medical diagnosis is paramount. This study looked at the current epidemiology of CL in Jordan. Clinically diagnosed 41 patients with CL were tested for the presence of* Leishmania* parasite using both Giemsa staining from skin scraps on glass slides and ITS1-PCR from samples blotted onto storage cards (NucleoCards®). Microscopically, 28 out of the 41 (68.3%) collected samples were positive for amastigotes, whereas the molecular ITS1-PCR amplification successfully identified 30 of the 41 samples (73.2%). Furthermore, PCR-RFLP analysis allowed species identification which is impossible microscopically. Of the 30 PCR positive samples, 28 were* Leishmania major* positive and the other two samples were* Leishmania tropica*. This indicates that* L. major* is the most prevalent species in Jordan and the two* L. tropica* cases originated from Syria indicating possible future* L. tropica* outbreaks. Diagnosis of CL based on clinical presentation only may falsely increase its prevalence. Although PCR is more sensitive, it is still not available in our medical laboratories in Jordan.

## 1. Introduction

Leishmaniasis threatens about 350 million people in 88 countries around the world [1]. It is believed that about 12 million people are currently infected with leishmaniasis, with about 1-2 million estimated new cases occurring every year [1]. The global incidence ranges of visceral leishmaniasis (VL) and cutaneous leishmaniasis (CL) are 0.2 to 0.4 cases and 0.7 to 1.2 million cases per year, respectively. More than 90% of the global VL cases occur in six countries, excluding Jordan. CL cases are more widely distributed and one-third of the cases occur in three epidemiological regions: the Americas, the Mediterranean basin, and western Asia from the Middle East to central Asia including Jordan [2].

Cutaneous leishmaniasis is endemic in many Middle Eastern countries such as Syria, Iraq, Saudi Arabia, and Jordan and is still regarded as a major health problem which requires international awareness [3]. Syria reported very high incidence of CL and although several Middle Eastern countries have well-established national control programs for controlling the spread of the vector (sand fly) and the treatment of the infected individuals, still the disease continues to spread especially in the past few years due to the human migration within this area which occurred due to the political instability in the region [3].

Cutaneous leishmaniasis due to* L. major* is an endemic disease in Jordan which is known as “Jericho boil.” Since 1985, outbreaks have appeared in areas where CL was previously unknown [4–7]. The occasional outbreaks of leishmaniasis in Jordan have occurred in endemic and nonendemic foci such as Aqaba, North Agwar, and South Shuneh. Based on an annual report released in 2009 by the Jordanian Ministry of Health, Alvar et al. [2] reported 227 CL cases per year between 2004 and 2008. However, this number is probably skewed due to a spike in 2007 where 354 cases were reported. The most recent Jordanian annual report identified a total of 2,560 CL cases throughout Jordan between 1994 and 2014 [8]. Severe underreporting of CL is suspected in Jordan, which impacts its eradication [9]. Many factors can lead to this underestimation, some of which include the self-healing nature of the disease, the lack of awareness of the physicians of the importance of disease notification, and the occurrence of the majority of the CL cases in endemic rural areas which have limited resources for treatment due to the scarcity of clinics.

In Jordanian rural areas, during outbreaks of CL, the diagnosis and treatment are usually made directly in the field based on epidemiological data and clinical presentation (i.e., lesion morphology). However, the definitive diagnosis of CL can be sometimes challenging even for expert clinicians, since the symptoms can vary and may be confused with other etiological agents. Thus, correct diagnosis is important for the selection of appropriate treatment and for the reduction of its complications [10]. The classical diagnosis of specimens that are taken from CL patients relies on the visualization of the parasite stage (amastigotes inside and outside macrophages) in Giemsa-stained smears. Microscopic examination of stained smear is rapid, cheap, and easy to perform but sometimes lacks sensitivity due to the generally low number of parasites in tissue samples [11], in addition to the need for experienced microscopist. Parasite culturing is more sensitive than microscopy but is time consuming, requires sophisticated laboratory setups, harbors the risk of contamination, and cannot identify species, as different species are morphologically indistinguishable [12].

Molecular tools using a number of molecular markers and polymerase chain reaction (PCR) protocols have been developed for the detection and identification of* Leishmania* [13–18]. For epidemiological investigation and clinical case management especially during outbreaks of leishmaniasis, the determination of the causative* Leishmania* species is extremely important and many of those molecular tools have already been used in different parts of the world including Jordan to differentiate species of* Leishmania* [19–21]. In the absence of molecular tools, geographical distribution, clinical presentation, and the species of sand fly vectors and animal reservoirs are commonly used to attempt to identify species, which is clearly inadequate, particularly where multiple species coexist [5, 17].

The present study is aimed at describing the epidemiology of human CL in Jordan using ITS1-PCR and RFLP in addition to Giemsa-stained smears microscopy. During the study period, we collected skin scrapping from 41 patients with suspected CL lesions. NucleoCards were used in the field to dry-preserve the genetic material for later molecular testing. Then NucleoCards ability to retain the genetic material and aid in CL diagnosis was compared to routine Giemsa-stain smear microscopy identification. The NucleoCards ability to hold parasites was not investigated in this study.

## 2. Materials and Methods

### 2.1. Study Population

The study participants were suspected patients with CL who were presented to the Ministry of Health Clinics in different parts of Jordan for the evaluation of skin lesions from early 2009 until late 2011. All patients gave their informed consent to participate in the study, which was reviewed by the institutional review board and approved by the Research Ethics Committee at Hashemite University and the Ministry of Health. A total of 41 patients thought to be infected with CL based on clinical examination only (i.e., size, number, location, and type of lesion) were recruited. These patients were interviewed to fill a demographic questionnaire, and then skin scrapings from the lesions were taken for the study.

### 2.2. Sample Collection for DNA Extraction and PCR

Samples from suspected* Leishmania* lesions were obtained from 41 patients. After removing any overlying scab or crust with moistened gauze, a DNA storage card, NucleoCards (740403.100, Macherey-Nagel, Germany), was gently pressed onto the moist ulcer base to allow the tissue fluid and scrapings to diffuse into the NucleoCards. At least two different samples/spots were collected on two different collection circles on the same card. The card was then allowed to air-dry for 30–60 min and stored at room temperature for subsequent DNA extraction. All cards were labeled with the patients' information and date of collection.

### 2.3. Sample Collection for Giemsa Staining

The same 41 patients were also screened by microscopy. After cleaning with topical antiseptic, material from the lesion (tissue scrapings) was collected from the ulcer base and border using a sterile lancet and spread on a glass slide. Slides were stained using a routine Giemsa staining procedure. All slides were viewed under oil immersion with 100x objective lens for confirmation of amastigotes stages inside and outside macrophages.

### 2.4. Extraction of DNA from NucleoCards

The DNA was extracted from the dry clinical samples (skin scrapings) collected on the NucleoCards using phenol-chloroform extraction manual procedure [22]. Briefly, two discs (5 mm) were punched out from each clinical sample blotted onto NucleoCards using a sterile blade and transferred to a sterile DNAase free tube containing 250 *μ*L cell lysis buffer (50 mM NaCl, 50 mM Tris, and 10 mM EDTA; pH 7.4), 2.5 *μ*L Triton X-100 (9002-93-1, Bio Basic Inc., Canada), and 1.26 *μ*L Proteinase K (20 mg/mL) (EO0491, Fermentas GmbH, Germany). The samples were incubated overnight at 60°C. The DNA was pelleted and dried at room temperature for 30 min and redissolved in 100 *μ*L of TE buffer (pH 8) (V6231, Promega, USA). The samples were kept at −80°C until assayed.

### 2.5. PCR Amplification

**Figure 1:**
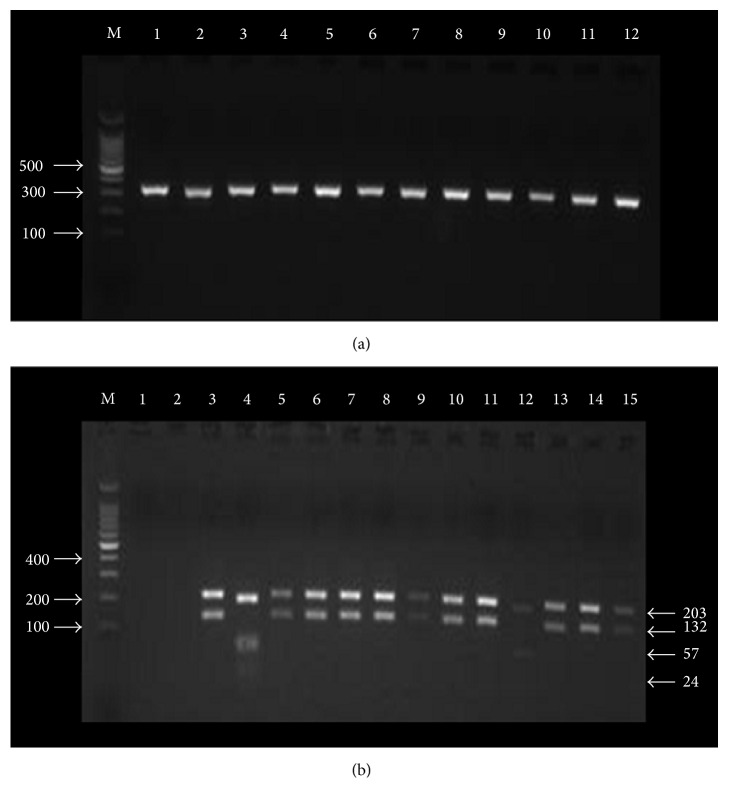
Representative pictures showing agarose gel electrophoresis (2%) of random PCR products (300–350 bp) which were extracted from the 30 positive* Leishmania* samples. Lane M: 100 bp DNA ladder. Lanes 1–12: PCR products randomly selected from 30 clinical samples (a) and (b) showing the digestion of amplified ITS1 regions for different* Leishmania* species with the restriction endonuclease Hae IIII. Lane M: 100 bp DNA ladder. Lane 1: negative control. Lane 2: healthy individual control. Lane 3:* L. major* positive control showing two bands (203 bp and 132 bp). Lane 4:* L. tropica* positive control showing three bands (185 bp, 57 bp, and 24 bp). Lanes 5, 6, 7, 8, 9, 10, 11, 13, 14, and 15: random samples for* L. major* detected in clinical samples. Lane 12:* L. tropica* detected in clinical samples.

Amplification of the ribosomal internal transcribed spacer 1 (ITS-1) gene was carried out using the LITSR (forward) (5′CTGGATCATTTTCCGATG-3′) and L. 5.8S (reverse) (5′-TGATACCACTTATCGCACTT-3′) primers [22]. Both primers and positive controls of* Leishmania* strains were kindly provided by Dr. Abdedelmajeed Nasereddin (Al-Quds University, Palestine). Amplification reactions were performed in a 25 *μ*L volume containing 1.5 *μ*L extracted DNA or control samples, 12.5 *μ*L of 2x PCR master mix (M7502, Promega, USA), 1.5 *μ*L of each primer (10 pmol/*μ*L), and 8 *μ*L nuclease-free water. Amplification was performed in a thermocycler (I Cycler, Bio-Rad, USA) according to El Tai et al. [22] with modification; thermal profile involved initial denaturation at 95°C for 2 min followed by 32 cycles consisting of denaturation at 95°C for 20 sec, annealing at 53°C for 30 sec, and extension at 72°C for 1 min. This was followed by a final extension at 72°C for 6 min. 5 *μ*L of the amplification products was assayed using 2% agarose gel electrophoresis (V3125, Promega, USA) at 120V for 45 min in 1x TBE buffer (0.045 M Tris-borate (H5131, Promega, USA) and 1 mM EDTA (H5031, Promega, USA)) after staining with of ethidium bromide (0.5 mg/mL) (H5041, Promega, USA) for 15 min. DNA bands of 300–350 bp that confirmed the presence of* Leishmania* DNA were visualized by ultraviolet transparent gel tray (UVI, EEC) and photographed using the IP-010-SD photo documentation system program (Vilber Lourmat, EEC). Each PCR run was routinely assessed against negative and positive controls. The two negative controls are clean NucleoCards without template DNA and NucleoCards spotted with healthy blood. Positive controls for* Leishmania* DNA included* L. tropica*,* L. major*,* L. infantum*, and* L. donovani*. After comparing to controls, an image was taken for a group of positive samples (Figure 1(a)).

### 2.6. Restriction Fragment Length Polymorphism (RFLP) Analysis of Amplified ITSI Products

To digest the PCR product, 15 *μ*L of the PCR products was mixed with 2 *μ*L of restriction enzyme buffer (10x), 0.2 *μ*L of BSA acetylated, 1.8 *μ*L of nuclease-free water, and 1 *μ*L of 10 U/*μ*L of the restriction enzyme* Hae* III as recommended by the supplier (R6175, Promega, USA). The mixture was incubated at 37°C for 2 hrs and then the restriction fragments were separated using 2% agarose gel electrophoresis as described above. An image was taken for a group of positive samples in addition to positive and negative controls after restriction (Figure 1(b)).

## 3. Results

### 3.1. Demographics

Briefly, samples were collected from patients diagnosed with CL based on clinical presentation attending various clinics in different parts of Jordan. The majority of the samples collected for this study were from Jordanians (83%), with six Syrians (15%) and one Egyptian. Detailed demographic data is shown in Table 1.

### 3.2. Comparison of Cutaneous Leishmaniasis Diagnosis Based on Clinical Presentation, Microscopy, and PCR Analysis

Based on clinical presentation, all 41 cases were suspected as CL patients. By microscopy,* Leishmania* amastigote stage was detected in 28 of these 41 samples (68.3%, 60.4–82.5%). The PCR procedure was used for the diagnosis and identification of* Leishmania* species for clinical samples saved on NucleoSave cards. Of the 41 samples, PCR amplicon could only be successfully extracted from 30 clinical samples and all of these were positive by PCR (72.3%, 59.6–87.5%). Figure 1(a) shows the PCR products (300–350 bp) from 12 randomly selected positive samples.

### 3.3. Species Identification by Restriction Analysis of the Amplified ITSI Region

RFLP analysis of the 30 PCR-positive clinical specimens with the restriction enzyme Hae III identified 28 samples infected with* L. major* and only two were infected with* L. tropica* (Figure 1(b)).

## 4. Discussion

The aim of this study is to use the ITS1-PCR and RFLP to describe the epidemiology of human CL and species identification of leishmaniasis in Jordan from dry-preserved samples on NucleoCards. The sensitivity of the PCR-RFLP technique was also compared with the clinical diagnosis and routine Giemsa-stain microscopy method. According to the study results, samples collected from CL suspected patient were positive for either* L. major* (28 samples) or* L. tropica* (2 samples) using the PCR-RFLP method. The median age of the study sample was 14 years and almost two-thirds of them were males (66%). The sample population was mainly Jordanians; however, there were some Syrians and one Egyptian. Based on the clinical diagnosis, most CL infections were in the usually exposed parts of the body such as upper and lower limbs and the face in patients who had resided in the same address for more than 10 years and nearby valleys. Despite living in endemic leishmaniasis area for many years, the majority of those patients showed little or no knowledge of the vector (sand flies) and, therefore, did not know how to protect themselves from CL infection (Table 1).

The PCR-RFLP method was found to be highly sensitive and specific in detecting samples that have* Leishmania* when compared to the Giemsa-stain microscopy method, which is congruent with previous reports [16, 23, 24]. Of the 41 samples, PCR amplicons could only be successfully extracted from 30 samples and all of these were positive by PCR. The other 11 samples did not have enough DNA concentration on the cards (<5 ng/*μ*L), hence the negative results, which indicate the need for more sample quantity during collection. A previous study reported the use of molecular detection of CL from lesions stored on NucleoCards filter paper (modified cards which are different from the normal filter paper) [14]. However, this study compared lesion scrapings for microscopic identification with blots on NucleoSave cards from lesions for its ability to diagnose CL and discriminate the infecting species based on dried DNA.

Obtaining clinical samples (lesions aspirates and scrapings) for CL diagnosis by microscopy and* in vitro* culturing in Jordan is the routine procedure. Nevertheless, it frequently causes pain and discomfort to patients and requires technical expertise [25, 26]. For existing and emerging foci of CL in Jordan, accurate diagnostic tools are required to detect parasites directly in clinical samples and distinguish all relevant species of* Leishmania*. The ITS1 sequence (300–350 bp depending on the species) was chosen in the present study as the target for our PCR assay for several reasons. Many recent studies have shown that ITS1-PCR followed by restriction fragment length polymorphism analysis is a suitable tool for diagnosing and identifying* Leishmania* species [17, 27]. The major advantage of ITS1-PCR is that species identification can be achieved by digesting the PCR product by just one restriction enzyme (Hae III) and this is sufficient to distinguish almost all medically important* Leishmania* species [17].

All cases of leishmaniasis reported from Jordanian patients are cutaneous and caused by the two species,* L. major* and* L. tropica* [9, 10]. The different* Leishmania* species are morphologically indistinguishable, but they can be differentiated by isoenzyme analysis, molecular methods, or monoclonal antibodies. Molecular tools have been developed for the differentiation of the pathogenic species of* Leishmania* in different parts of the world [19–21]; however, recently, just one study evaluated using such a PCR technique in Jordan [28] but still molecular based assay is not a readily available diagnostic technique in our medical laboratories despite the fact that species discrimination is important for epidemiological and clinical reasons.

Until recently, relatively little information has been available on the epidemiology of CL and the causative species in different parts of Jordan. Leishmaniasis due to* L. major* species is considered hyperendemic in the Jordan Valley and Wadi Araba regions, with some areas exhibiting 80% positivity using* Leishmania* skin testing among exposed populations in hyperendemic areas and an infection rate of 5.5% and 23% in the vector and reservoir hosts, respectively [29]. There appears to be an evolutionary divergence between the* L. major* zymodemes isolated from the Jordan Valley and the Jordanian plateau [30]. These regions are separated by a mountainous region where no cases of* L. major* have been reported. In the North where the biotope is more Mediterranean and the land is rocky, only* L. tropica* infections have been reported [31, 32]. This area has a semi-Mediterranean climate and is about 600 meters above sea level. No evidence for the presence of rodent hosts of* L. major* (*P. obesus*) was found [33].

In the North of Jordan,* L. tropica* has been reported as a causative agent for CL, whereas* L. major* has been reported in all other areas of the country. Those findings are based on geographical distributions, data collected by the Health Department and local studies, clinical presentations, vectors, and animal reservoirs. The Jordan Valley is home to endemic areas with very high infection rates. In the hyperendemic area of Swaimeh, 100% of individuals over 5 years old were found positive in a* Leishmania* skin test survey in 1992, which is similar to our results where the average age of patients was 18.3 years [34]. In the same survey, higher infection rates (72.4%) are recorded in males than females (27.6%) in all age groups which is also congruent with our findings where 66% and 34% of the suspected CL patients were males and females, respectively. It was also reported that CL is more prevalent in children under 5 years (24%) than in those older than 50 (8%), as we also noted in this study which may be explained by the natural vulnerability of this population [34].

The available epidemiological evidence indicated that all forms of CL in Jordan are mainly zoonotic diseases [35], where the most prevalent* Leishmania* species in Jordan is* L. major* which is responsible for 75% of the cases [35, 36]. Large areas of the country, particularly the Southern and Eastern regions, correspond to desert biotopes in which* L. major*, a zoonotic infection, is maintained by the reservoir host* Psammomys obesus, Meriones libycus*, and the sand fly vector* Phlebotomus papatasi*. Cases from* L. tropica* are rare and reported from the northern regions of Jordan. The suspected vector for* L. tropica* is the sand fly* Phlebotomus sergenti* and the reservoir host is still unknown but evidence has suggested that canines or hyraxes, which are present in all* L. tropica* foci in Jordan, are both suspected to be the reservoir hosts [1].

In regions such as the Middle East including Jordan, patients suffering from leishmaniasis often live in remote and isolated low socioeconomic areas with limited access to clinical care and education [10]. When they become infected with* Leishmania* they are often undiagnosed patients and when they present to their local medical center, there are insufficient diagnostic tools and expertise. We believe that using sample storage cards (NucleoCards) is very effective and helpful in such regions. The material can be collected on the NucleoCards which can be then mailed via normal local mail to the central diagnostic facility where molecular tools and skilled specialist are available to properly diagnose the material in a relatively short period of time, allowing better patient management and treatment.

Cutaneous leishmaniasis is well known to be an endemic disease not only in Jordan but also in neighboring countries such as Syria [37]. In the year 2012 an official figure reported 52,982 confirmed cases of CL in Syria [38]. With the political instability in Syria, millions of Syrian refugees entered the neighboring countries of Lebanon, Turkey, and Jordan [38]. In Jordan, only 20% of the refugees reside in the Al Zaatari camp while the remaining 80% reside within the Jordanian population in major cities. The extensive displacement of the Syrian population also increased the incidence of the vector-borne disease within Syria and its spread into neighboring countries [38]. In this study we found that the majority of CL is caused by* L. major* (28 cases) in a mixture of nationalities of Jordanians (22), Syrians (5), and one Egyptian. There were only two cases of CL that were caused by* L. tropica*. One of them is a Syrian and the other is a Jordanian who indicated a previous travel to Syria. As a result, it is obvious that future outbreaks of CL caused by* L. tropica* more than* L. major* might be unavoidable in Jordan especially in areas that have a high number of refugee residents. This requires higher alert level by the Ministry of Health in Jordan and proactive role in preventing outbreaks and the spread of CL. Early detection, proper diagnosis, and treatment are the gold standard recommendations. Using ITS1-PCR to identify the infecting* Leishmania* species in clinical samples could be the future routine test that needs to be adopted in Jordan.

## Figures and Tables

**Table 1:** Major characteristics of the study participants (*n* = 41).

Characteristic (*N*)				
Gender	M (27) 66%	F (14) 34%		
Age (years)	0.5–73	Average 18.3	Median 14	Mode 14
Education	None (21)	<grade 10 (13)	>grade 10 (7)	
Nationality	Jordanian (34)	Syrian (6)	Egyptian (1)	
Infected body parts	Facial (13)	Upper limbs (23)	Lower limbs (16)	Trunk (2)
Stage of infection	Cured (7)	Papule (2)	Ulcerated (31)	Ulcerated + papule (1)
Number of lesions	1–8 (average 1.99)	Average M 1.99	Average F 1.97	
Microscopic results	Negative (13)	Positive (28)		
PCR results (*Leishmania* species)	*L. major* (28)	*L. tropica* (2)	Negative (11)	
Treatment	Yes (37)	No (4)		
Residency in current address (years)	<5 (13)	5–10 (3)	>10 (25)	
Outdoor activities	Yes (30)	No (11)		
Travelled outside Jordan in the past year	Yes (3)	No (38)		
Sand fly knowledge	Yes (9)	No (32)		
House type	Cement (33)	Hair tent (8)		
Protections from sand flies	None (25)	Some (16)		
Living nearby valley, stream, or farm	Yes (38)	No (3)		

*N*: number; M: male; F: female; some protection: window sieve, insecticides, and/or insect repellent.
